# Redox Regulation of Megakaryocyte Differentiation and Platelet Biogenesis

**DOI:** 10.3390/antiox15030352

**Published:** 2026-03-11

**Authors:** Hyunmin Chung, Eunju Shin, Taeho Park, Hanseul Jeong, Haiyoung Jung, Ok-Nam Bae, Ji-Yoon Noh

**Affiliations:** 1 Aging Convergence Research Center, Korea Research Institute of Bioscience and Biotechnology (KRIBB), Daejeon 34141, Republic of Korea; hyunmin508@kribb.re.kr (H.C.); ejshin@kribb.re.kr (E.S.); luiziana06@kribb.re.kr (T.P.); beulah1241@kribb.re.kr (H.J.); haiyoung@kribb.re.kr (H.J.); 2 Department of Functional Genomics, Korea University of Science & Technology (UST), Daejeon 34113, Republic of Korea; 3 College of Pharmacy, Institute of Pharmaceutical Science and Technology, Hanyang University, Ansan 15588, Republic of Korea; onbae@hanyang.ac.kr

**Keywords:** megakaryocyte, reactive oxygen species, differentiation, redox signaling, platelet yield, cell therapy

## Abstract

Pathological accumulation of reactive oxygen species (ROS) is implicated in several diseases, including cancer, cardiovascular diseases, and aging. However, ROS play essential roles in cellular functions, including proliferation, differentiation, and immune responses, at physiological levels. In megakaryocytes, the cells responsible for producing platelets, ROS exert context-dependent effects, either promoting or impairing maturation depending on developmental stage and subcellular localization. In this review, we summarize current evidence demonstrating that balanced ROS signaling is required throughout megakaryocyte development. Further, we discuss how the source and timing of ROS generation determine their distinct stage-specific functions, and the role of ROS dysregulation in defective platelet production in conditions such as aging, inflammation, and hematopoietic stress. We further highlight the importance of redox regulation for efficient in vitro platelet manufacturing. Although stem cell-derived platelets hold great promise for addressing global platelet shortages, current systems produce significantly fewer platelets than are found naturally. We propose that limited understanding and poor control of ROS dynamics contribute to limited platelet yield and quality. By viewing ROS as tunable biological signals rather than solely as harmful byproducts, we emphasize redox modulation as a practical and actionable approach to enhance platelet biogenesis and support the development of next-generation platelet therapies.

## 1. Introduction

Reactive oxygen species (ROS) are generated during normal physiological metabolism and immune defense. They accumulate from exogenous sources, including radiation, environmental pollutants, cigarette smoke, iron salts, and toxins. Intracellular ROS are produced through multiple enzymatic pathways, including NADPH oxidase (NOX) complexes, xanthine oxidases, amine oxidases, nitric oxide synthases, myeloperoxidase, peroxisomes, and arachidonic acid metabolism by lipoxygenases and cyclooxygenases [1,2], and the subcellular localization of these enzymes, antioxidants, and redox targets is tightly regulated within cells. In addition, physiological processes such as oxidative protein folding in the endoplasmic reticulum and fatty acid β-oxidation in peroxisomes contribute to intracellular ROS generation [3]. Together, antioxidant systems and redox-sensitive mechanisms maintain ROS homeostasis and prevent excessive oxidative stress.

Megakaryopoiesis is the developmental process of platelet biogenesis, which produces approximately 100 billion platelets per day, corresponding to an average output of ~3000 platelets per megakaryocyte (MK). During differentiation, MKs migrate from relatively low-oxygen regions of the bone marrow toward higher oxygen areas. Concurrently, mitochondrial biogenesis increases during early megakaryopoiesis, resulting in elevated levels of mitochondrial ROS (mtROS). Despite these observations, the precise mechanisms by which intracellular ROS levels and oxidative stress responses regulate MK maturation remain incompletely understood [4].

Recently, several countries have faced shortages of platelet transfusions owing to aging populations, low birth rates, and pandemics [5]. Substantial efforts, therefore, have been directed toward overcoming donor-dependent platelet transfusion therapy and developing in vitro platelet production processes from human stem cells [6,7,8]. This approach became more feasible after Megakaryon Corporation, building on the pioneering work of Koji Eto’s group at Kyoto University, successfully produced more than 10^11^ platelets for autologous transfusion and reported the results of a phase 1 clinical trial (iPLAT1) [9]. Although platelet biogenesis from induced pluripotent stem cells (iPSCs) and cord blood (CB)-derived hematopoietic stem cells (HSCs) can be recapitulated in vitro, MKs have not yet been studied at physiological levels [10,11]. Current estimates indicate that in vitro systems yield only 10–100 platelets per MK, and it remains unclear whether rare “super MKs” producing ~3000 platelets exist in vivo or whether most CD41^+^CD42b^+^ MK-like cells generate only 10–100 platelets per MK. Therefore, understanding the in vivo microenvironment and its effect on MKs is essential for improving the yield of in vitro platelet production.

In this review, we discuss redox-sensitive signaling pathways and their functional consequences during megakaryopoiesis. Furthermore, we summarize the current understanding of ROS regulation in cell therapy development processes, with the goal of elucidating the role of ROS in megakaryopoiesis in vitro.

## 2. Context-Dependent Roles of ROS in Megakaryopoiesis

### 2.1. ROS Biology: Types, Sources, and Regulatory Mechanisms

Major cellular ROS include the superoxide anion (O_2_^•−^, often abbreviated as O_2_^−^), hydrogen peroxide (H_2_O_2_), hydroxyl radical (•OH), and reactive nitrogen species (RNS). O_2_^−^ is generated primarily through electron leakage to molecular oxygen, mainly at electron transport chain (ETC) complexes I and III during oxidative phosphorylation. In addition to mitochondrial sources, O_2_^−^ can arise as secondary byproducts of cellular metabolism or from specialized enzymes such as NOX, which are localized in the plasma membrane or endomembranes and play key roles in immune defense and cellular signaling. The NOX family includes NOX1-5, DUOX1, and DUOX2, all of which transport electrons across membranes to generate O_2_^−^ and downstream ROS [1,12]. NOX1, NOX2, and NOX4, together with their regulatory subunits, have been detected in human stem and progenitor cells isolated from peripheral blood [13,14]. Studies in MKs and platelets have demonstrated expression of NOX1, NOX2, and NOX4 in both humans and mice, with NOX1 and NOX4 being particularly abundant in primary murine MKs, and NOX2 also detected in the human MEG-01 megakaryocytic cell line [15].

The mitochondrial ETC is a central component of ATP production. During oxidative phosphorylation, ATP synthesis is accompanied by ROS generation through electron leakage and oxygen reduction, producing O_2_^−^ or H_2_O_2_ [16,17]. H_2_O_2_—generated through the activity of mitochondrial or cytosolic superoxide dismutases (SODs)—is relatively stable and diffusible and functions as a signaling molecule by oxidizing specific cysteine residues in target proteins. In the presence of ferrous iron (Fe^2+^), H_2_O_2_ generates •OH via the Fenton reaction. These radicals are highly reactive and interact with biomolecules, including DNA, lipids, and proteins. Excessive lipid peroxidation leads to ferroptosis, which is inhibited by glutathione peroxidase 4 (GPx4), which detoxifies lipid ROS using cellular glutathione (GSH) [18,19,20,21]. Recent studies have shown that GPx4 expression protects differentiating MKs with iron overload–induced ferroptosis and supports effective platelet recovery. In platelets, mitochondrial SOD2 reduces mtROS-driven phosphatidylserine exposure, thrombin generation, and age-related arterial thrombosis; therefore, SOD2 serves as a key antioxidant enzyme for maintaining platelet redox homeostasis [22,23].

The nuclear factor erythroid-derived 2-like 2 (NRF2) pathway acts as a master regulator of antioxidant gene expression. Under basal conditions, NRF2 is bound by Kelch-like ECH-associated protein 1 (KEAP1) and undergoes ubiquitination and proteasomal degradation. Elevated ROS levels modify cysteine residues on KEAP1, enabling NRF2 translocation to the nucleus and binding to antioxidant response elements (AREs) [24,25]. In MK, NRF2 both cooperates and competes with the platelet transcription factor NF-E2 p45 to regulate the expression of cytoprotective genes. As MKs mature, NRF2 activity declines, leading to reduced antioxidant gene expression, increased ROS accumulation, and enhanced expression of platelet-specific genes, thereby promoting megakaryocytic maturation [26]. The transcription factors FOXO1 and FOXO3 are redox-sensitive and promote antioxidant gene expression. FOXO3 plays a critical role in maintaining ROS balance in HSCs and protecting against oxidative DNA damage through regulation of base excision repair mechanisms. Additionally, in MK progenitors, FOXO3a and its downstream target p27(Kip1) contribute to thrombopoietin (TPO)-induced proliferation, linking FOXO signaling to the early stages of megakaryopoiesis [27,28,29].

### 2.2. Pro-Differentiation Roles of ROS in MK Development

ROS promote megakaryopoiesis through extrinsic (microenvironmental) and intrinsic (cell-autonomous) pathways. Within the bone marrow, elevated oxygen levels enhance MK maturation and platelet production. This effect is shaped by the physiological oxygen gradient, in which the low O_2_ levels in the osteoblastic niche help to maintain HSC quiescence, whereas the high oxygen tension near the sinusoidal regions promotes MK maturation and progression toward thrombopoiesis [30,31,32].

Consistent with this microenvironmental influence, studies using leukemia cell lines (K562 and Dami) treated with phorbol 12-myristate 13-acetate (PMA) or endocannabinoids have demonstrated that intracellular ROS accumulation correlates with MK differentiation. In K562 cells, PMA-induced differentiation is accompanied by increased ROS levels, upregulation of MK-specific markers (CD41, CD42a, and CD61), enhanced endomitosis leading to polyploidization, and increased cell size. In these models, pharmacological inhibition of NOX significantly reduces ROS generation and attenuates PMA-induced MK features, indicating that ROS produced following NOX activity is a major upstream link between PMA stimulation and megakaryocytic differentiation in K562 cells [33,34].

At the intracellular level, NOX enzymes are the major source of ROS during megakaryopoiesis, particularly p22^phox^-dependent NOX enzymes. RNA interference studies have shown that p22^phox^-dependent NOX activity directly drives ROS production in differentiating MKs. Accordingly, inhibition of ROS generation using antioxidants such as N-acetyl-L-cysteine (NAC), Trolox, and quercetin or the NOX inhibitor diphenyliodonium (DPI) significantly suppresses MK differentiation [2]. In mouse bone marrow cultures, NOX1 and NOX4 are upregulated following TPO treatment. Furthermore, inhibition of NOX activity results in impaired endomitosis, leading to an accumulation of immature, low-ploidy MKs and a marked decrease in polyploid MK populations. Purified NOX-inhibited MKs exhibit reduced levels of G1-phase cyclin E, a key regulator of polyploidy; restoration of cyclin E expression rescues the polyploidy suppressed by NOX inhibition, underscoring the critical role of NOX-derived ROS in endomitosis and early MK maturation [15,35].

Beyond NOX-dependent cytosolic ROS, mtROS generated downstream of TPO signaling function as metabolic signals that promote progenitor differentiation toward the megakaryocytic lineage, particularly during the late stages of megakaryopoiesis. Upon binding to its receptor, MPL, TPO not only activates canonical kinase pathways but also induces rapid metabolic reprogramming in HSCs and MK progenitors, shifting the ATP production mechanism from glycolytic metabolism toward oxidative phosphorylation. This transition increases mitochondrial ETC activity and mtROS generation, which enhances megakaryocytic differentiation potential in vitro and myeloid/MK-biased reconstitution in vivo, indicating that TPO-activated mtROS act as positive signals for MK fate decisions [36]. These metabolically derived mtROS are rapidly dismutated by SOD2 to H_2_O_2_, which can diffuse to the cytosol and modulate the activity of redox-sensitive transcription factors and signaling intermediates, such as GATA1, NF-E2, MAPK, and PI3K/AKT, thereby promoting MK-specific gene expression programs [2,32,37].

The natural peroxisome proliferator-activated receptor gamma (PPARγ) ligand 15-deoxy-Δ^12,14^-prostaglandin J_2_ (15d-PGJ_2_) enhances platelet release from MKs while concomitantly increasing intracellular ROS levels. Inhibition of the antioxidant gene heme oxygenase-1 (HO-1) using protoporphyrin IX (SnPPIX) further potentiates the effect of 15d-PGJ2 on MK differentiation by amplifying ROS accumulation. These observations support the concept that elevated ROS levels positively regulate platelet biogenesis during the late stages of megakaryopoiesis [38,39]. Clinically, HO-1 deficiency in humans is associated with elevated platelet counts and increased systemic oxidative injury, reinforcing the physiological importance of redox homeostasis in platelet production and vascular integrity [40,41]. Specifically, terminal MK maturation, particularly proplatelet formation, is accompanied by a marked increase in mtROS, which function as signaling mediators to fine-tune redox-dependent pathways during MK differentiation. During late megakaryopoiesis, mitochondrial remodeling events—particularly mitochondrial fission—are associated with enhanced mtROS production, which promotes cytoskeletal reorganization and proplatelet elongation [4].

Together, these findings establish a clear stage-specific distinction in ROS function during megakaryopoiesis: NOX-dependent ROS act primarily during early MK lineage commitment and polyploidization, whereas mtROS serve as the physiological triggers for terminal morphological reorganization and cytoplasmic fragmentation that culminate in platelet release. This stage-dependent separation highlights the necessity for precise spatial and temporal control of distinct ROS to achieve complete and efficient megakaryopoiesis.

### 2.3. Inhibitory Effects of Excessive ROS Accumulation on Megakaryocyte Differentiation

Excessive ROS accumulation disrupts mitochondrial homeostasis and redox balance, thereby impairing MK differentiation and platelet production. In autophagy-deficient MKs, elevated ROS levels block differentiation and cause defective thrombopoiesis, thus necessitating intact redox control mechanisms during MK maturation [42]. Interestingly, in certain contexts, reducing intracellular ROS levels instead enhances MK differentiation. This suggests a complex biphasic relationship in which both insufficient and excessive ROS negatively affect megakaryopoiesis. Consistent with this model, treatment with docosahexaenoic acid (DHA) or arachidonic acid (AA) reduces ROS production and apoptosis in CB-derived CD34^+^ cells, thereby increasing platelet yield in vitro. These polyunsaturated fatty acids promote MK proliferation and differentiation, as evidenced by significantly increased expression of CD41, CD61, and CD42b. Moreover, DHA-treated hematopoietic precursors display improved engraftment in NOD/SCID mice, accompanied by increased human CD61 levels, suggesting that lower ROS levels are particularly beneficial during the early stages of MK differentiation from HSCs [43,44]. Similarly, pharmacological or phytochemical agents that attenuate oxidative stress, such as the NF-κB inhibitor parthenolide, enhance MK maturation and platelet generation [45].

Multiple in vitro studies further demonstrate that maintaining redox balance is essential for efficient MK maturation and platelet formation. In immortalized megakaryocyte progenitor cell lines (imMKCLs) and CB-derived MKs, excessive ROS accumulation disrupts mitochondrial homeostasis and reduces platelet yield, whereas pharmacological suppression of ROS restores differentiation efficiency. In parallel, genetic or pharmacological modulation of KCNN4, a calcium-activated potassium channel involved in mitochondrial function, alters intracellular ROS levels and MK maturation, reinforcing the importance of tightly controlled oxidative signaling during thrombopoiesis [46,47]. In the bone marrow microenvironment, excessive ROS accumulation in endothelial progenitor cells (EPCs) impairs MK differentiation following allogeneic HSC transplantation. Prolonged isolated thrombocytopenia (PT), a frequent post-transplant complication, is characterized by dysfunctional EPCs exhibiting elevated ROS levels, increased apoptosis, and reduced angiogenic activity. Treatment with the ROS scavenger NAC restores EPC function, enhances megakaryopoiesis, and improves graft recovery [48]. Consistently, clinical trials have shown that prophylactic oral NAC administration safely and effectively prevents poor graft function and PT by promoting dynamic reconstitution of bone marrow ECs and HSCs [49]. In addition, leukemic stem cells exhibit aberrantly high ROS levels that disrupt normal differentiation programs, further reinforcing that excessive oxidative stress within the hematopoietic niche hinders effective thrombopoiesis [50].

Collectively, these findings indicate that while physiological ROS levels are required for early lineage commitment, excessive oxidative stress impairs MK maturation. Partial attenuation of ROS, therefore, restores proper differentiation and platelet formation, emphasizing the necessity for balanced redox control during megakaryopoiesis [51].

## 3. Redox Signaling Pathways Governing Megakaryocyte Fate Decisions

### 3.1. ROS as Signaling Mediators in Megakaryopoiesis

**Figure 1:**
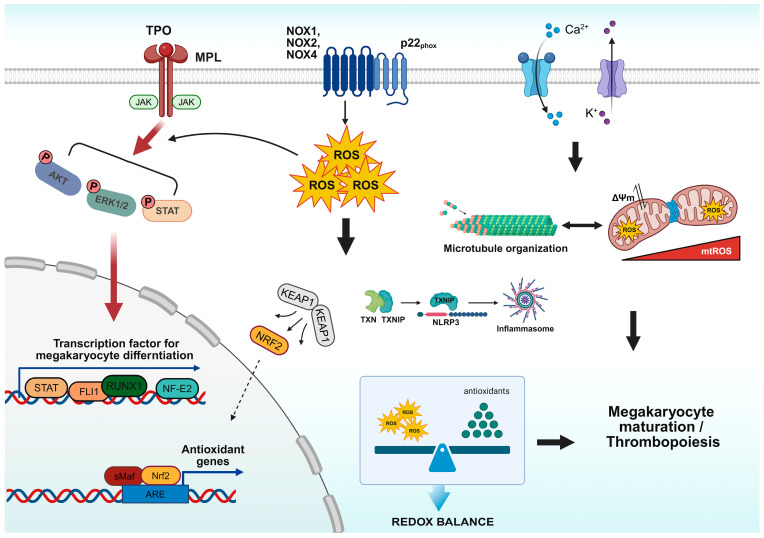
Redox signaling networks in megakaryocyte (MK) differentiation. MK maturation and platelet biogenesis are regulated by context-specific redox signaling. Thrombopoietin (TPO) binds to its receptor MPL, activating the JAK–STAT, PI3K–AKT, and ERK1/2 pathways, which in turn induce the expression of transcription factors, such as RUNX1, FLI1, NF-E2, and STAT, thereby promoting MK differentiation. NADPH oxidases (NOX) generate cytosolic ROS that further potentiate these signaling cascades, whereas mitochondrial ROS (mtROS) accumulation drives cytoskeletal remodeling and proplatelet formation during terminal maturation. During proplatelet formation, mtROS—coupled with mitochondrial fission and K^+^–Ca^2+^ flux—directly regulate microtubule cytoskeletal organization through ROS-sensitive tubulin polymerization, enabling the structural polarization and dynamic rearrangements required for efficient proplatelet elongation. The thioredoxin-interacting protein (TXNIP) complex and KEAP1–NRF2 axis maintain redox homeostasis by modulating antioxidant gene expression. Under excessive oxidative stress, ROS-TXNIP-NLRP3 inflammasome signaling promotes MK terminal differentiation through caspase-1-mediated apoptosis, facilitating platelet shedding. The graphical abstract was created using BioRender.com. Abbreviations: TPO: Thrombopoietin; JAK: Janus kinase; STAT: Signal transducer and activator of transcription; PI3K: Phosphatidylinositol 3-kinase; ERK: Extracellular signal-regulated kinase; RUNX1: Runt-related transcription factor 1; FLI1: Friend leukemia integration1; NF-E2: Nuclear factor, erythroid 2; NOX: NADPH oxidase; ROS: Reactive oxygen species; mtROS: Mitochondrial ROS; TXN: Thioredoxin; TXNIP: Thioredoxin-interacting protein; KEAP1: Kelch-like ECH-associated protein 1; NRF2: Nuclear factor erythroid 2-related factor 2.

Understanding ROS dynamics is critical for defining MK differentiation fate. During megakaryopoiesis, intracellular ROS levels must be coordinated in a stage-specific manner, making it essential to consider not only overall ROS levels but also their sources, species, and signaling mechanisms. In this section, we describe how ROS function as signaling mediators during megakaryocytic lineage differentiation from hematopoietic progenitor cells (Figure 1).

#### 3.1.1. Redox Control of Transcription Factor Networks in Megakaryocytes

Fetal liver–derived primary MKs exhibit ROS-dependent regulation of platelet gene expression, coordinated primarily by the transcription factors NF-E2 p45 and Nrf2 [26]. Comparative analysis of CD41^+^DCFDA^low^ and CD41^+^DCFDA^high^ populations revealed that increased ROS accumulation enhances the expression of platelet-associated genes, including thromboxane synthase (Txas), glycoprotein VI (Gp6), P-selectin (Selp), and Slamf1. During megakaryopoiesis, upregulation of NF-E2 p45 increases intracellular ROS levels, which, in turn, promote platelet gene expression while concomitantly suppressing Nrf2 target genes, thereby sustaining ROS accumulation in maturing MKs. In contrast, p45-null mice exhibit severe thrombocytopenia and impaired terminal MK differentiation, accompanied by dysregulated stress-responsive gene expression [52,53]. NAD(P)H:quinone oxidoreductase 1 (NQO1), an Nrf2-induced antioxidant enzyme involved in ROS detoxification, is dynamically required during megakaryopoiesis. The balance between NF-E2 p45 and Nrf2 activity, therefore, determines intracellular ROS levels in MKs. As MKs mature, p45 predominates over Nrf2, suppressing cytoprotective gene expression and permitting the ROS accumulation required for platelet gene expression and release [54,55].

ROS-induced MK differentiation is further coordinated by the transcription factor FLI-1 [56]—an ETS transcription factor—that directly interacts with RUNX-1 and synergistically activates MK-specific gene expression, including that of TPO receptor MPL. The interaction between RUNX-1 and FLI-1 is differentiation-dependent and regulated by dephosphorylation of FLI-1 at serine 10. This phosphorylation-dependent control of FLI-1 activity plays a critical role in terminal MK maturation [57]. Together, these findings indicate that ROS tune MK gene expression largely by shifting the balance among NF-E2, NRF2, and FLI-1/RUNX-1, thereby coupling redox status to lineage-specific transcriptional programs.

#### 3.1.2. ROS-Regulated Signaling Pathways in Megakaryopoiesis

At the molecular level, ROS activate multiple redox-sensitive signaling pathways that are essential for MK proliferation and differentiation. Upon stimulation of human hematopoietic cells with growth factors such as TPO, intracellular ROS levels rapidly increase, initiating a cascade of downstream signaling events [51]. H_2_O_2_ stimulation enhances tyrosine phosphorylation of several signaling proteins, including the common βc subunit, STAT5, c-KIT, SHC adapter protein, and the protein tyrosine phosphatase SHP-1 in the MO7e MK cell line [58]. A key mechanism by which ROS propagate intracellular signaling is through inhibition of protein phosphatases such as PP1α, resulting in sustained activation of the PI3K/AKT pathway in leukemic cells [32]. In parallel, the MEK–ERK1/2 pathway represents a critical ROS-responsive signaling axis during MK differentiation. PMA-induced ERK activation in K562 and HEL cells is abolished by ROS inhibition, and pharmacological blockade of MEK–ERK1/2 signaling using U0126 or PD186141 leads to increased CD34 expression, reduced GPIb expression, and a leftward shift in ploidy distribution. These findings highlight the requirement for ERK signaling in MK polyploidization and maturation [59]. ROS also regulate stress-related kinase pathways in MKs. Dopamine stimulation elevates intracellular ROS levels, leading to activation of p38 MAPK and c-Jun NH2-terminal kinase (JNK), both of which are essential for MK proliferation and differentiation [60]. In addition, ROS modulates inflammatory signaling by activating transcription factors such as NF-κB. H_2_O_2_ directly activates NF-κB, thereby regulating genes involved in cell survival and differentiation. Upstream receptors—including toll-like receptors, tumor necrosis factor receptors, and interleukin (IL) receptors—converge on NF-κB during megakaryopoiesis. Notably, activity of the IkappaB kinase complex (IKK) is elevated during early differentiation stages and declines as MKs progress to thrombopoiesis, facilitating controlled cell death and platelet release [61]. Thus, by integrating growth factor, stress, and inflammatory signaling through interconnected kinase and phosphatase pathways, ROS function as a central regulatory signal to MK cell differentiation and maturation.

#### 3.1.3. Redox Regulation of Microtubule Organization in Thrombopoiesis

Proplatelet formation from mature MKs requires extensive remodeling of the microtubule cytoskeleton, which provides the structural basis for elongation and platelet release. Tubulin, the major microtubule component, contains multiple cysteine residues (12 in α-tubulin and 8 in β-tubulin) that are highly sensitive to oxidative modification. Physiological levels of ROS promote tubulin polymerization and dynamic turnover, whereas excessive ROS oxidize these cysteines, induce disulfide cross-linking, and destabilize the microtubule lattice, leading to defective proplatelet formation [62,63,64,65]. The influence of ROS on microtubule dynamics extends directly to proplatelet initiation, which is coordinated by potassium channel signaling and mitochondrial function. The KCNN4 channel, selectively upregulated at the onset of platelet biogenesis, maintains intracellular potassium homeostasis by promoting K^+^ efflux during late MK maturation. KCNN4 activity preserves mitochondrial membrane potential (ΔΨm) and ROS balance; conversely, inhibition or knockdown of KCNN4 leads to excessive ROS accumulation and profound disruption of microtubule organization, thereby preventing the symmetry-breaking events required for proplatelet outgrowth. Microscopic analysis demonstrates that KCNN4 dysfunction causes asymmetric tubulin distribution and disorganized microtubule arrays, a phenotype recapitulated by direct ROS elevation using tert-butyl hydroperoxide (TBHP), confirming a causal role of ROS dysregulation in suppressing microtubule organization [47].

Beyond potassium homeostasis, calcium signaling acts in parallel with ROS to fine-tune the cytoskeletal organization during proplatelet formation. Calcium mobilization from intracellular stores and extracellular influx via store-operated calcium entry (SOCE) channels activate downstream pathways that promote MK adhesion, contractility, and proplatelet elongation [66]. Intracellular Ca^2+^ flux is closely coupled to mitochondrial activity and ROS production, forming a dynamic feedback loop that stabilizes microtubule organization. Inhibition of key Ca^2+^ signaling regulators, such as casein kinase 2β (CK2β) or N-methyl-D-aspartate (NMDA) receptor subunits, disrupts tubulin polymerization and impairs proplatelet formation, underscoring the interdependence of Ca^2+^ homeostasis, mtROS, and microtubule integrity [67,68].

Collectively, these studies demonstrate that redox signaling integrates ionic homeostasis with cytoskeletal remodeling during megakaryopoiesis. Coordinated regulation of potassium efflux through KCNN4 and calcium influx via SOCE channels maintains ΔΨm and ROS balance, thereby preserving the redox-sensitive microtubule cytoskeleton required for proplatelet formation. This controlled K^+^–Ca^2+^–ROS interplay not only preserves cytoskeletal integrity but also orchestrates the spatial polarization and dynamic rearrangements essential for efficient proplatelet formation and subsequent platelet biogenesis.

#### 3.1.4. Thioredoxin-Based Redox Systems in Megakaryocyte Development

The thioredoxin-1 (TXN1) system plays a critical role in maintaining redox hematopoiesis and megakaryopoiesis. TXN1 is essential for normal blood cell production in adult mice, as conditional deletion of TXN1 results in lethality within 30 days of tamoxifen induction, accompanied by severe weight loss and pancytopenia [69]. Thioredoxin-interacting protein (TXNIP) further regulates cellular and mitochondrial redox balance by interacting with TXN and other redox-sensitive proteins. TXNIP-deficient mice (Txnip^−/−^) develop progressive thrombocytopenia beginning at 4–5 months of age, which worsens with aging. During ex vivo megakaryopoiesis, Txnip^−/−^ megakaryocyte precursors (MKPs) remain small and display reduced expression of MK-specific markers, indicating impaired differentiation. Notably, Txnip^−/−^ MKPs exhibit reduced mtROS levels associated with altered AKT signaling. This redox imbalance is accompanied by a metabolic shift toward elevated glycolysis and increased glucose uptake to sustain ATP production. Transcriptomic analysis revealed enrichment of oxidative stress- and apoptosis-related gene signatures among differentially expressed genes in Txnip^−/−^ MKPs compared with wild-type controls [70].

In addition to its role in redox–metabolic coupling, TXNIP has been proposed to act as an oxidative stress sensor that links TXN redox control to NOD, LRR-, and pyrin domain-containing protein 3 (NLRP3) inflammasome signaling. Upon ROS-dependent dissociation from TXN, TXNIP can bind NLRP3 and facilitate inflammasome activation with downstream IL-1β maturation [71]. A recent study reported that the ginsenoside metabolite compound K promoted megakaryocytic differentiation in K562 (and MEG-01) cells while concomitantly upregulating an NLRP3 inflammasome gene program, suggesting that inflammasome tuning can intersect with MK differentiation and apoptosis-linked platelet release [72].

Collectively, these observations indicate that the loss of TXNIP disrupts redox-metabolic coupling. TXNIP loss may also rewire ROS-sensitive TXNIP–NLRP3 inflammasome signaling in MK precursors, leading to age-dependent exhaustion of megakaryopoiesis and a reduced pool of terminally mature MKs capable of responding to thrombocytopenic challenge [70]. Therefore, the TXN/TXNIP axis represents a critical redox checkpoint linking metabolic homeostasis to MK differentiation and platelet biogenesis.

### 3.2. Insights from Animal Models: Redox Dysregulation in Pathophysiological Megakaryopoiesis

#### 3.2.1. NOX1,2 Deficiency Reveals Redox Control of Platelet Biogenesis and Function

The NOX isoforms NOX1 and NOX2 play distinct and context-dependent roles in platelet redox signaling. NOX2 is the catalytic core of the NOX2 complex and is best known for its role in oxidative stress and intracellular signaling. Genetic deficiency in NOX2 causes chronic granulomatous disease (CGD), a condition characterized by impaired ROS generation and dysregulated immune responses. Early studies reported that platelets from patients with CGD exhibit defective ROS production, secretion, and activation, including reduced thrombin-induced phosphorylation of Syk and PLCγ, suggesting a functional role in platelet signaling [73,74]. In contrast, NOX1 is primarily engaged downstream of G protein–coupled receptor (GPCR) agonists such as thrombin and thromboxane A_2_, where it mediates rapid ROS production that amplifies platelet secretion and integrin activation. Delaney et al. further demonstrated that NOX2 plays a dominant role downstream of adhesion receptors, particularly immunoreceptor tyrosine-based activation motif (ITAM)–containing receptors such as GPVI, contributing to thrombus formation at sites of vascular injury under high shear conditions [75]. However, Walsh et al. reported that NOX1, rather than NOX2, is the primary source of GPVI-dependent ROS generation and that neither isoform is strictly required for GPVI-induced platelet aggregation. Instead, both NOX1 and NOX2 contribute to collagen-induced thrombus formation under arterial shear stress. In this model, NOX1 deficiency reduces ROS and thromboxane A_2_ production following collagen-related peptide (CRP) stimulation, resulting in delayed thrombus formation in vivo, while tail bleeding times remain normal in both models, suggesting that NOX1 drives collagen-dependent thrombotic responses, whereas NOX2 fine-tunes ROS output under mechanical stress [75,76].

Despite these distinctions, the precise contribution of NOX2 in platelet function remains unclear. Sonkar et al. showed that NOX2-deficient mice display normal platelet aggregation, secretion, adhesion, and arterial thrombosis, concluding that NOX2 is largely dispensable for platelet activation and thrombus formation, with redox compensation likely mediated by NOX1 or mitochondrial oxidases [77]. Consistently, Xu et al. demonstrated that NOX2 activation is driven by outside-in integrin signaling and shear forces, enhancing thrombus growth under arterial flow while remaining non-essential for physiological hemostasis [78].

Collectively, these findings support a stimulus- and context-dependent model in which NOX1 primarily mediates GPCR-driven, soluble agonist–dependent platelet activation, whereas NOX2 contributes to collagen- and shear-dependent thrombotic signaling with partial redundancy. This functional division reconciles discrepancies among experimental models and highlights how receptor engagement and vascular mechanics determine NOX isoform utilization. Notably, systemic oxidative stress also influences megakaryopoiesis, as stress-induced NOX1 activation in bone marrow MKs increases platelet output—an effect reversed by the NOX inhibitor apocynin [79].

Together, these observations demonstrate that NOX-dependent redox signaling spans multiple stages of thrombopoiesis, linking MK differentiation to platelet activation within both local and systemic redox environments.

#### 3.2.2. Aging-Associated Redox Imbalance and Altered Megakaryopoiesis

Physiological aging is associated with elevated intracellular ROS levels in HSCs, driven by increased oxidative phosphorylation, reduced antioxidant enzyme expression, and cumulative oxidative damage [80]. Aging is accompanied by progressive HSC dysfunction and altered lineage output, a process known as hematopoietic aging. During aging, a hallmark change in the HSC compartment is a shift toward myeloid-biased HSCs with concomitant reduction in lymphoid-biased output. Within this broader myeloid skewing, platelet/MK-primed subsets (often operationally defined as CD41^+^ HSCs or p-HSCs) also expand with age [81,82,83]. Notably, CD41^+^ HSCs increase in response to cellular stressors such as age-associated elevated ROS [84]. Mechanistic insights into this process—linking redox dysregulation to increased CD41^+^ HSCs during aging—have emerged from studies in TXNIP-deficient mice. Global transcriptomic profiling of Txnip^−/−^ MKPs revealed enrichment of oxidative stress- and apoptosis-associated gene signatures [70]. CD41^+^ HSCs exhibit a MK/platelet-biased transcriptional program and are primed toward accelerated differentiation [83]. Elevated ROS levels and chronic inflammation during aging expand this biased HSC subset and reinforce lineage commitment through redox-sensitive pathways, including p38 MAPK and ERK–ETS1. Emerging evidence further suggests that increased IL-27 receptor α (IL27Rα) signaling in aged HSCs may synergize with redox imbalance to exacerbate myeloid and megakaryocytic bias. Although IL27Rα induction is primarily inflammation-driven rather than ROS-dependent, its convergence with oxidative stress likely contributes to the exaggerated platelet production and HSC exhaustion during aging [85,86].

#### 3.2.3. Superoxide Dismutase Dysregulation and Age-Related Thrombotic Risk

SODs provide critical protection against oxidative damage in platelets that express cytosolic SOD1 and mitochondrial SOD2, which together limit O_2_- accumulation. In young platelets, mtROS production remains low and SOD2 activity is relatively dispensable. In contrast, aged platelets exhibit markedly elevated mitochondrial O_2_- levels and increased mitochondrial pro-oxidants, accompanied by decreased activity of other antioxidant systems, including glutathione peroxidase (GPx-1), peroxiredoxin (Prdx-6), and catalase. Recent studies demonstrate that mtROS contribute to age-associated thrombosis and that endogenous SOD2 protects against platelet-dependent thrombin generation and arterial thrombosis during aging. Treatment with avasopasem manganese (GC4419), a pharmacological SOD mimetic, reduces mitochondrial and cellular ROS levels, suppresses procoagulant platelet formation, and attenuates arterial thrombosis in aged mice [87,88].

#### 3.2.4. Mitochondrial Transfer Within the Bone Marrow Niche: Redox Implications

Recent research revealed that healthy MKs can transfer mitochondria to mesenchymal stem cells (MSCs) via connexin 43 (Cx43)–dependent gap junctions. This unidirectional mitochondrial transfer contributes to the generation of platelets with a low-energy, resting phenotype characterized by enhanced LYN activation. In contrast, MSCs exhibit limited capacity to donate mitochondria back to MKs. Experiments using MSCs from conditional Cx43-deficient mice demonstrate that Cx43 gap junctions are required for mitochondrial transfer from MKs to MSCs. In pathological settings such as sickle cell disease, dysfunctional MSCs display markedly reduced Cx43 expression and fail to accept mitochondria from MKs. Consequently, sickle cell disease–derived MKs retain excess mitochondria, leading to elevated ROS production and a shift toward hyperactivated, procoagulant platelet phenotypes [89,90]. Emerging evidence from mitochondrial transfer studies suggests that intercellular mitochondrial exchange not only alters metabolic and bioenergetic profiles in recipient cells but also modulates ROS dynamics [91]. In several models, transferred mitochondria have been reported to either alleviate oxidative stress by enhancing antioxidant capacity or influence redox signaling pathways in recipient cells, indicating that mitochondrial transfer may reshape the redox landscape within the bone marrow niche and thereby potentially influence megakaryopoiesis.

#### 3.2.5. Organellar Sources of ROS in Megakaryocyte Differentiation

Alterations in organelles contribute to ROS dysregulation and thereby influence hematopoiesis. Peroxisomes play key roles in free-radical detoxification, bile acid synthesis, and long-chain fatty acid catabolism. In a peroxisome-deficient mouse model (PEX1-Gly844Asp knock-in), elevated ROS levels were observed in hematopoietic stem and progenitor cells (HSPCs) and MSCs, accompanied by increased stem cell factor (SCF) production from MSCs. This model exhibits expanded HSPC, lymphocyte, neutrophil, and platelet populations, indicating that oxidative stress can bias hematopoietic lineage output toward thrombopoiesis [92].

Mitochondrial complex II dysfunction further illustrates the impact of organellar ROS on hematopoiesis. In mice harboring a missense mutation in succinate dehydrogenase complex subunit C (*Sdhc*^V69E^), HSCs display increased ROS levels, DNA damage, myeloid skewing, leukopenia, macrocytic anemia, and thrombocytosis. As complex II functions at the intersection of the TCA cycle and ETC, its dysfunction leads to succinate accumulation and excessive mtROS production, thereby regulating hematopoietic homeostasis [93].

## 4. Stepwise Regulation of Redox Signaling During In Vitro Platelet Production

In modern cell therapy manufacturing, redox regulation has emerged as a critical strategy for improving the yield, functionality, and stability of differentiated cellular products, including platelets. In vitro production of platelets from stem cells is a promising approach for transfusion medicine and regenerative applications, yet achieving clinically relevant yields and consistent functional quality remains challenging. As megakaryopoiesis and platelet biogenesis are tightly regulated by ROS and redox signaling in vivo, redox biology offers a useful framework for optimizing platelet production in vitro. Accordingly, culture strategies that modulate intracellular ROS levels, such as controlled oxygen tension and defined antioxidant buffering, are being increasingly explored to preserve physiological cell function and support scalable production. In this section, we discuss current limitations in in vitro platelet production, review redox-related principles regulating stem and progenitor cell maintenance, and highlight emerging redox-based design strategies for improving platelet biogenesis.

### 4.1. Current Strategies and Limitations of Stem Cell-Derived Platelet Biomanufacturing

In vitro megakaryopoiesis and platelet production have been achieved using embryonic stem cells (ESCs), iPSCs, and adult HSCs. Human pluripotent stem cells (hPSCs) are particularly attractive because they can be genetically modified to enhance self-renewal and serve as expandable precursors for MKs. A notable example is the imMKCL, which expresses doxycycline (DOX)-inducible c-MYC, BMI1, and BCL-XL [94]. imMKCLs can be robustly expanded in culture for more than five months, including after cryopreservation. Suppression of c-MYC, BMI1, and BCL-XL expression triggers terminal differentiation and the production of CD42b^+^ platelets. Based on this platform, a turbulence-controllable bioreactor was developed to enable clinical-scale platelet generation, which achieved production volumes of up to 8 L [95]. This advancement enabled the first phase I clinical trial of stem cell-derived platelet transfusions (iPLAT1). The transfusion was well tolerated, with no major adverse events, although no significant post-infusion increase in platelet count was observed [9].

In parallel, several groups have focused on generating expandable MK progenitors capable of terminal differentiation through transcription factor modulation. Ghevaert and colleagues demonstrated that overexpression of GATA1, TAL1, and FLI1 in ESCs biases differentiation toward the megakaryocytic lineage, generating so-called forward-programmed MKs (fopMKs) [96]. These reprogrammed MKs could proliferate in culture for over 90 days while maintaining MK purity, marker expression (CD41/CD42a), and platelet production. Starting from 1 × 10^6^ iPSCs, this approach yielded approximately 2 × 10^11^ MKs and an estimated total of 1 × 10^12^ platelets. GATA1 is a central transcription factor governing erythroid and MK differentiation, and loss-of-function mutations or reduced GATA1 expression in humans and mice result in the accumulation of immature MKPs [97]. This finding led to the development of the GATA1-null MK–erythroid progenitor cell line (G1ME2), generated by incorporating a DOX-inducible Gata1 knockdown system into mouse ESCs. G1ME2-derived MKs produce approximately 40 platelets per MK, approximating physiological output [98].

Despite these technological advances, both platelet yield and functional quality remain suboptimal. Each platelet typically carries five to eight mitochondria, and intact mitochondrial function with appropriate ΔΨm is essential for normal platelet activation and responsiveness [99,100]. Moreover, megakaryopoiesis from CB-derived CD34^+^ HSPCs exhibits dynamic changes in mitochondrial fission and function, suggesting that ROS production and redox signaling are tightly regulated during differentiation [4]. Current strategies to improve platelet yield—including genetic manipulation, bioreactor optimization, and cell line engineering—are likely to perturb intracellular redox states. In addition, redox signaling in vitro differs substantially from that within the native bone marrow microenvironment. To date, the specific impact of redox modulation on stem cell–derived platelet production, including effects on platelet yield, mitochondrial integrity, and functional quality, has not been systematically investigated. Understanding and deliberately controlling redox signaling during in vitro megakaryopoiesis, therefore, represents an underexplored yet potentially transformative avenue for advancing platelet cell therapies.

### 4.2. Redox Requirements for Stem Cell Maintenance and Hematopoietic Differentiation

In scalable in vitro platelet production, genetically engineered PSCs and their derived expandable hematopoietic progenitors serve as renewable starting materials [94]. Establishing such systems requires stable genetic modification, long-term expansion, and maintenance of differentiation potential through multiple culture transitions before terminal MK maturation and platelet release. As redox state influences cell cycle control, stress adaptation, and lineage commitment during these stages, ROS regulation should be considered a critical process. Accordingly, this section discusses redox-related principles governing PSC maintenance and differentiation (Section 4.2.1) and hematopoietic cell systems (Section 4.2.2). These examples provide a conceptual framework for considering how redox regulation may support MKP expansion and stable cell line establishment in scalable platelet manufacturing.

#### 4.2.1. Metabolic and Redox Regulation in Pluripotent Stem Cells (PSCs) During Expansion and Engineering

PSCs are predominantly glycolytic, possess functionally active mitochondria, and exhibit marked metabolic plasticity [101,102]. Physiological hypoxia (∼3–5% O_2_) is frequently used to maintain an undifferentiated state, although reported effects on growth and pluripotency markers vary across studies [103]. Importantly, under in vitro conditions, ROS levels are not uniformly low in PSCs: when ROS readouts are normalized (e.g., per cell volume or protein), overall ROS levels can be comparable between PSCs and differentiated cells [104]. Consistent with this metabolic flexibility, iPSC reprogramming involves metabolic rewiring with suppression of oxidative phosphorylation, which can reshape mitochondrial ROS tone [105]. Moreover, in hPSCs, ROS varies across the cell cycle, and antioxidant-driven ROS lowering impairs S-phase initiation/progression with reduced accumulation of cyclin A and geminin [106]. Taken together, these findings support treating oxygen tension and antioxidant dosing as tunable process variables—avoiding both chronic oxidative stress and ROS over-quenching—during long-term PSC maintenance and upstream expandable progenitor stages [107,108,109].

Three-dimensional organoid/aggregate systems better approximate in vivo niches by generating oxygen gradients that stabilize hypoxia-inducible factor 1-alpha (HIF-1α) and activate hypoxia-responsive programs (e.g., Vascular Endothelial Growth Factor (VEGF) and growth/inflammatory signaling) [110,111]. For instance, in retinal and other organoid models, early hypoxia supports progenitor proliferation and preserves immature transcriptional programs, whereas later phases require adequate oxygen to sustain mitochondrial metabolism, ROS signaling, and terminal maturation [112,113,114]. Consistent with observation, stirred-tank bioreactor expansion of hiPSC-derived cardiomyocytes under controlled mild hypoxia reduces intracellular ROS generation while increasing proliferative capacity and genomic stability, illustrating how 3D hydrodynamics plus oxygen control can improve scalable cell manufacturing [115,116]. In PSC-to-hematopoietic differentiation toward MKs, 3D (e.g., embryoid body-based) formats can prolong CD34^+^ progenitor maintenance, delay MK differentiation kinetics, and expand MKP/mature MK pools that yield functional platelets [117]. Bone marrow organoids further demonstrate that niche-level oxygen/redox cues shape MK states in both physiology and disease contexts such as myelofibrosis [118].

#### 4.2.2. Redox Regulation in Suspension-Based Hematopoietic Culture Systems

**Figure 2:**
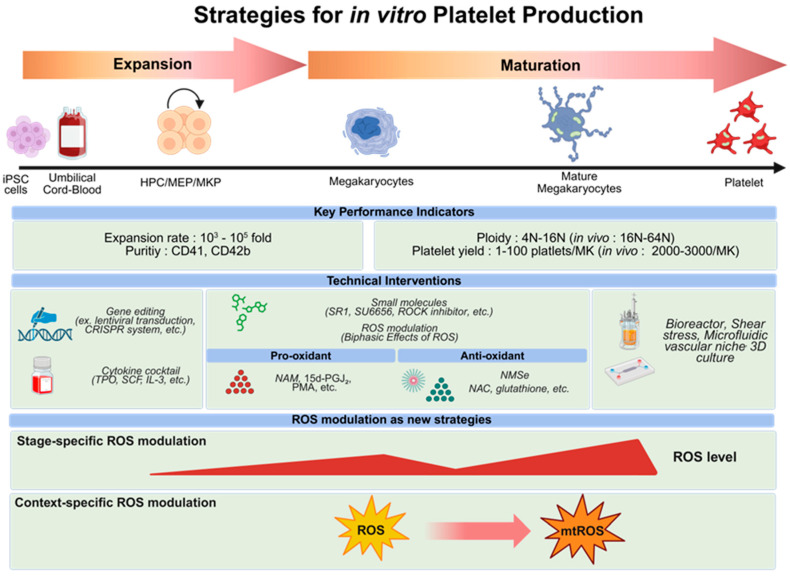
In vitro platelet production: stage-specific interventions and ROS-based optimization. Schematic overview of in vitro platelet production from human stem cells, such as iPSC or UCB-derived HSCs, highlights currently used performance indicators and emerging ROS-modulating strategies. During the expansion phase, hematopoietic progenitors (HPC/MEP/MKP) undergo extensive proliferation (>10^3^–10^5^ fold) under cytokine cocktails (e.g., TPO, SCF, and IL-3) and gene editing or immortalization systems (e.g., lentiviral transduction [94], CRISPR system [127]). In the differentiation phase, megakaryocytes (MKs) increase in size and polyploidy (4N–16N, versus 16N–64N in vivo) and generate limited platelet yields (1–100 platelets per MK). Small-molecule modulators such as SR1 [128], SU6656 [129], and ROCK inhibitors [130], together with stage-specific ROS modulation—including pro-oxidant cues (e.g., NAM [4], 15d-PGJ_2_ [38,39], PMA [33,34]) and antioxidant buffering (e.g., NMSe [131], NAC [125], glutathione [132])—can further enhance MK maturation efficiency. Advanced bioreactors [95], microfluidic systems mimicking vascular shear stress [133] and 3D culture [117] improve proplatelet formation and platelet release. The lower section illustrates a conceptual framework in which stage-specific and context-dependent ROS modulation serves as a potential strategy for optimizing MK differentiation. Controlled redox tuning—maintaining mild ROS fluctuations during early expansion and promoting transient mitochondrial ROS accumulation at later maturation stages—may represent a promising approach to recapitulating physiological thrombopoiesis in vitro. The graphical abstract was created using BioRender.com. Abbreviation: MK: Megakaryocyte; ROS: Reactive oxygen species; HPC: Hematopoietic progenitor cell; MEP: Megakaryocyte-erythroid progenitor; MKP: Megakaryocyte progenitor; iPSC: induced pluripotent stem cells; UCB: Umbilical cord blood; TPO: Thrombopoietin; SCF: Stem cell factor; IL: Interleukin; ROCK: Rho-associated coiled-coil protein kinase; NAM: Nicotinamide; PMA: Phorbol 12-myristate 13-acetate; NMSe: Selenium-containing nanomicelles; NAC: N-acetyl-L-cysteine.

In vitro platelet generation from stem cells proceeds through a stepwise procedure from hematopoietic progenitor cells (HPCs) to MK–erythroid progenitors (MEPs), MKPs, mature MKs, and finally platelets (Figure 2). As this process passes through intermediate progenitor states, the quality of early populations is a major determinant of downstream MK maturation and platelet output. Such stages are often defined primarily by surface markers; however, cell quality attributes—particularly redox status—have been less emphasized. Although ROS generation is an unavoidable consequence of cellular respiration, its magnitude varies by metabolic pathway: anaerobic glycolysis produces minimal ROS, whereas mitochondrial respiration generates substantially higher levels. Excessive ROS accumulation disrupts redox homeostasis, leading to loss of quiescence and diminished self-renewal of HSCs [119]. HSCs are multipotent adult stem cells that maintain low intracellular ROS levels to preserve self-renewal capacity and quiescence. ROS can influence cell fate through oxidative post-translational modifications, particularly via oxidation of cysteine residues in redox-sensitive proteins. In HSCs, quiescence is tightly regulated through redox-dependent control of signaling pathways involving p53, NF-κB, and intracellular thiol balance [120,121].

As oxygen tension is a major upstream driver of redox state in HSC culture, HIF-1α serves as a central regulator linking hypoxia signaling to metabolic programming and ROS control in the bone marrow niche. By supporting hypoxic/glycolytic programs, HIF-1α can limit mtROS accumulation and is stabilized in HSCs under steady-state niche conditions, where it supports long-term repopulating capacity and protects against senescence and cell death. Conversely, loss of HIF-1α is associated with elevated oxidative stress, impaired quiescence, and reduced long-term regenerative potential [122,123].

From a manufacturing perspective, once early progenitors are generated, they must be expanded without the loss of their differentiation competence, a challenge conceptually mirrored in clinically approved, ex vivo–expanded chimeric antigen receptor (CAR)-T cell therapies. In CAR-T cells, chronic stimulation during expansion can induce mitochondrial dysfunction and mtROS-associated exhaustion, compromising proliferative capacity and persistence. In a chronic stimulation model, NAC supplementation during culture reduced ROS generation and improved bioenergetic fitness, shifting transcriptional programs away from terminal exhaustion toward progenitor-like/self-renewal states [124,125]. Clinical trials have also explored NAC supplementation to improve CAR-T persistence, although this targets the post-infusion milieu rather than the ex vivo expansion phase [126].

### 4.3. Redox Modulation as a Design Principle for Megakaryopoiesis and Platelet Biogenesis

In scalable platelet manufacturing, achieving high yield alone is not sufficient. As platelets are anucleate and highly sensitive to environmental stress, preventing hyperactivation while preserving functional stability is important. In this context, ROS regulation emerges as a key factor linking cellular expansion, differentiation, and functional integrity. In this section, we focus on studies related to MK maturation and platelet biogenesis in which redox modulation has been examined. Although relatively few studies have examined ROS as a primary engineering variable, they provide useful insights on how cell culture systems may influence platelet yield and quality. Together, these examples suggest that redox control can be considered as a practical design element in stepwise in vitro thrombopoiesis (Figure 2).

#### 4.3.1. Antioxidant and Pro-Oxidant Modulation

Several antioxidant compounds including vitamins C and E, α-tocopherol, trolox, β-carotene, selenium, resveratrol, flavonoids, NAC, and glutathione have been evaluated for their effects on platelet generation. Among these, resveratrol has been consistently shown to suppress aberrant platelet activation while preserving physiological platelet function. Specifically, resveratrol reduced in vitro thrombus formation, platelet adhesion, aggregation, and TXA_2_ production in both healthy individuals and patients with diabetes mellitus. Mechanistically, resveratrol attenuates platelet activation by inhibiting glucose metabolism and reducing the activity of key metabolic enzymes, including hexokinase, G-6-P-dehydrogenase, aconitase, and isocitrate dehydrogenase, thereby limiting cellular energy production. In parallel, resveratrol decreases thiobarbituric acid-reactive substance (TBARS) levels, confirming its efficacy in reducing lipid peroxidation [134].

In contrast, mild pro-oxidant stimulation, such as treatment with nicotinamide or phenethyl isothiocyanate, can enhance proplatelet formation, highlighting the dual and context-dependent roles of ROS signaling. Consistent with this concept, mitochondria-targeted ROS scavengers, including mitoTEMPO, markedly reduce proplatelet formation, indicating that mtROS, rather than cytosolic ROS, function as positive regulators of cytoskeletal remodeling and platelet release. Yoshihara et al. demonstrated that mtROS levels are dynamically regulated during MK maturation. Early-stage round MKs exhibit low mtROS levels and a highly tubular mitochondrial network, despite detectable mitochondrial recruitment of the fission mediator dynamin-related protein 1 (DRP1). As MKs enter the intermediate stage at the onset of thrombopoiesis, mtROS levels rise sharply and mitochondria become markedly fragmented, indicating that DRP1-dependent fission becomes functionally engaged at this stage to drive proplatelet formation. Pharmacological inhibition of mitochondrial fission using the DRP1 inhibitor Mdivi-1 reduces intermediate-stage MKs and impairs proplatelet formation, whereas expression of a phosphomimetic DRP1 mutant (S616D) enhances mitochondrial fission and promotes platelet release. Similarly, overexpression of a deacetylase-inactive SIRT3 mutant, which limits MnSOD-dependent detoxification and elevates mtROS levels, augments proplatelet formation. Conversely, inhibition of cytosolic ROS generation using NOX inhibitors (apocynin or VAS2870) does not affect platelet release, reinforcing that mtROS, rather than NOX-derived ROS, provide the critical local signal that initiates thrombopoiesis [4].

Collectively, these findings indicate that precise redox tuning—rather than global ROS suppression—is required to balance MK differentiation and proplatelet formation. Pharmacological modulators, such as NOX inhibitors (e.g., setanaxib), glutathione synthesis inhibitors (buthionine sulfoximine), and thioredoxin reductase inhibitors, can reshape redox signaling and influence lineage commitment [104,135].

#### 4.3.2. Genetic and Pharmacological Modulation for Enhanced Megakaryocyte Progenitor Production

A recent study demonstrated that a VGM cocktail—comprising HES7 overexpression, the HDAC inhibitor MC1568, and a GABA agonist [136]—increased MKP production efficiency by nearly 90%, accompanied by marked increases in polyploidization (8N–32N) and expression of mature MK markers (CD41^+^/CD42b^+^). This high-efficiency MKP induction has been validated across HSPCs from multiple donors. The resulting MKPs exhibit enhanced proliferative capacity, remain viable for up to 51 days in prolonged culture, and show improved maturation into MKs. Transcriptomic analyses revealed activation of NF-κB, MAPK, cGMP-PKG, and PI3K-AKT pathways, with JAK2-STAT3 signaling identified as a central mediator, many of which are closely associated with redox regulation [136,137]. Importantly, transfusion of VGM-induced MKPs into thrombocytopenic mice resulted in detectable platelet release into the circulation. Together, these findings indicate that the VGM cocktail promotes platelet production by efficiently expanding MKP populations, offering a promising strategy for in vitro platelet regeneration in clinical applications.

#### 4.3.3. Bioreactor Systems and Turbulent Flow

Bioreactor systems that recapitulate vascular shear forces and oxygen gradients markedly enhance platelet biogenesis. The recognition of turbulence as a physiological regulator of thrombopoiesis in vivo has enabled its translation into turbulence-controllable bioreactors. By identifying turbulent energy as a determining parameter, platelet production has been successfully scaled to 8 L, yielding ~100 billion platelets from hiPSC-derived imMKCLs, satisfying clinical requirements. hiPSC platelets exhibit hallmarks of bona fide human platelets, including circulation competence and hemostatic function following transfusion in animal models [95].

Consistent with these findings, Taylor–Couette (TC) bioreactor systems that apply controlled shear via inner cylinder rotation (~1500 rpm) achieve substantially higher platelet yields than static cultures. This improvement likely reflects shear-induced physiological signaling, including enhanced mtROS generation in regions of elevated oxygen tension. However, recent studies indicate that platelet production efficiency depends not only on achieving sufficient shear stress or turbulent kinetic energy, but also on the uniformity of their impact across the MK population. Systems in which individual MK experience highly variable flow conditions are less efficient, even when overall turbulence parameters are comparable [138].

Computational fluid dynamics analyses further demonstrate that uniform, periodic exposure of individual MK to turbulent forces—often achieved through circular flow trajectories—is critical for efficient platelet release. These configurations ensure consistent mechanical and redox cues at the single-cell level over time, thereby promoting platelet production [139]. Collectively, these findings underscore that successful large-scale bioreactor design must integrate fluid dynamics, mechanical stress distribution, and redox microenvironment control to optimize both the yield and functional quality of stem cell–derived platelets.

## 5. Conclusions

Redox regulation has emerged as a central determinant of megakaryopoiesis and platelet biogenesis. Distinct ROS sources—primarily NOX-derived cytosolic ROS and mitochondria-derived mtROS—govern MK lineage commitment, polyploidization, proplatelet formation, and ultimately platelet function in a stage- and context-dependent manner. In this review, we explored how NOX activity promotes G1–S cell-cycle progression during the early endomitotic stages of megakaryopoiesis, and how mtROS accumulation, coupled to TPO-induced metabolic reprogramming, biases cells toward enlarged and mature MKs. However, the precise interplay between NOX-derived cytosolic ROS and mtROS during megakaryopoiesis, as well as the quantitative thresholds at which each source effectively promotes differentiation, remains incompletely understood. Current evidence indicates that increases in both ROS sources within a physiological range are required for efficient MK development, whereas excessive or chronically dysregulated ROS accumulation triggers oxidative stress–mediated dysfunction and impaired maturation.

Notably, terminal megakaryopoiesis, encompassing proplatelet formation and platelet release, appears to be regulated by substantially different redox dynamics. Emerging evidence indicates that elevated ROS levels during late-stage maturation can disrupt microtubule organization, impair cytoskeletal remodeling, and reduce platelet yield. These observations suggest that compartmentalized and/or transient attenuation of ROS signaling may be required for efficient terminal differentiation. Consequently, the rational design of in vitro platelet production systems will require stage-specific redox programming rather than uniform antioxidant application throughout differentiation.

Despite substantial advances in the understanding of ROS-regulation-mediated differentiation, the existing literature has some limitations. Most of the available data were derived from mouse or immortalized MK models, whereas ROS regulation of primary MKs in humans remains poorly characterized. Moreover, the lack of compartment-specific probes, redox biosensors and quantitative standards for “physiological” ROS levels limited our ability to define the optimal redox range for each developmental stage. Furthermore, although mtROS elevation accompanies human MK maturation in vitro, the threshold at which it promotes differentiation instead of impairing it remains undefined. Additionally, robust real-time assays to assess redox competency in developing MKs are not yet available. Ultimately, these questions can be resolved by combining MK studies with functional platelet production and testing how regulating redox affects yield and platelet quality under scalable culture conditions.

In summary, understanding the temporal and localization contexts in which ROS act during MK differentiation needs to be addressed in the future. Integrating precise redox control into platelet manufacturing—through temporally regulated oxygen gradients, metabolic reprogramming, and source-specific ROS modulation—is a promising approach for future studies. Defining quantitative redox thresholds and applying them in scalable stem cell-derived platelet production will be key steps toward translating redox biology into clinically relevant, high-yield, and functionally robust platelet manufacturing platforms.

## Data Availability

No new data were created or analyzed in this study.

## Abbreviations

The following abbreviations are used in this manuscript:

•OH	Hydroxyl radical
15d-PGJ_2_	15-deoxy-Δ^12,14^-prostaglandin J_2_
AA	Arachidonic acid
AREs	Antioxidant response elements
CAR	Chimeric antigen receptor
CGD	Chronic granulomatous disease
CK2β	Casein kinase 2β
CRP	Collagen-related peptide
Cx43	Connexin 43
DHA	Docosahexaenoic acid
DOX	Doxycycline
DPI	Diphenyliodonium
DRP1	Dynamin-related protein 1
EBs	Embryoid bodies
EPCs	Endothelial progenitor cells
ESCs	Embryonic stem cells
ETC	Electron transport chain
FLI1	Friend leukemia integration 1 transcription factor
fopMKs	Forward-programmed megakaryocytes
G1ME2	GATA1-null megakaryocyte–erythroid progenitor cell line
GLUT	Glucose transporter
GP6	Glycoprotein VI
GPCR	G protein–coupled receptor
GPx4	Glutathione peroxidase 4
GSH	Glutathione
H_2_O_2_	Hydrogen peroxide
HIF1α	Hypoxia-inducible factor-1α
HO-1	Heme oxygenase-1
hPSCs	Human pluripotent stem cells
HSCs	Hematopoietic stem cells
IKK	IkappaB kinase complex
IL	Interleukin
imMKCLs	immortalized megakaryocyte progenitor cell lines
iPSCs	induced pluripotent stem cells
ITAM	Immunoreceptor tyrosine-based activation motif
JNK	c-Jun NH2-terminal kinase
KEAP1	Kelch-like ECH-associated protein 1
MK	Megakaryocyte
MKPs	Megakaryocyte precursors
MPL	Myeloproliferative leukemia protein
MSCs	Mesenchymal stem cells
mtROS	Mitochondrial ROS
NAC	N-acetyl-L-cysteine
NLRP3	NOD, LRR-, and pyrin domain-containing protein 3
NMDA	*N*-methyl-*D*-aspartate
NMSe	Selenium-containing Nanomicelles
NOX	NADPH oxidases
NQO1	NAD(P)H:quinone oxidoreductase 1
NRF2	Nuclear factor erythroid-derived 2-like 2
O_2_^−^	superoxide anion
PDK	Pyruvate dehydrogenase kinase
PMA	Phorbol 12-myristate 13-acetate
PPARγ	Peroxisome proliferator-activated receptor gamma
PPIX	Protoporphyrin IX
PRDX	Peroxiredoxin
PSCs	Pluripotent stem cells
PT	Prolonged isolated thrombocytopenia
ROS	Reactive oxygen species
RUNX1	Runt-related transcription factor 1
SCF	Stem cell factor
SOCE	Store-operated calcium entry
SOD	Superoxide dismutase
STB	Stirred-tank bioreactor
TBARS	Thiobarbituric acid-reactive substance
TBHP	Tert-butyl hydroperoxide
TPO	Thrombopoietin
TXAS	Thromboxane synthase
TXN1	Thioredoxin-1
TXNIP	Thioredoxin-interacting protein
ΔΨm	Mitochondrial membrane potential
